# Effects of psychosocial interventions on children affected by parental HIV/AIDS: a meta-analysis on depression and anxiety

**DOI:** 10.1186/s12889-019-7806-x

**Published:** 2019-11-27

**Authors:** Peilian Chi, Shan Zhao, Chen Zhang, Xiaoming Li, Yan Guo, Xiuyun Lin, Hongfei Du

**Affiliations:** 1 Department of Psychology, University of Macau, Macau, China; 2 School of Nursing, University of Rochester, Rochester, USA; 3 Department of Health Promotion, Education, and Behavior, Arnold School of Public Health University of South Carolina, Columbia, USA; 4 Department of Biostatistics and Epidemiology, School of Public Health, Sun Yat-sen University, Guangzhou, China; 5 Institute of Developmental Psychology, School of Psychology, Beijing Normal University, Beijing, China; 6 Department of Psychology, Guangzhou University, Guangzhou, China; 7 Social and Health Psychology Research Center, Guangzhou University, Guangzhou, China

**Keywords:** Children, Parental HIV/AIDS, Psychosocial interventions, Meta-analysis, Depression, Anxiety

## Abstract

**Background:**

Children orphaned by parental AIDS or those of parents with HIV infection demonstrate many negative mental health outcomes. Different types of psychosocial interventions have been conducted to improve the psychological well-being of these children. The efficacy of these psychosocial interventions has been reviewed and synthesized recently (Skeena et al., Vulnerable Child Youth Stud 12:91-116, 2017), but not quantified.

**Method:**

This study therefore adopted meta-analytic approach to quantify the efficacy of the existing psychosocial interventions on depressive and anxiety symptoms in children affected by parental HIV/AIDS. Eight intervention studies—four randomized controlled trials (RCT) and four pre–post intervention trials—were included.

**Result:**

In general, psychosocial interventions could effectively reduce anxiety or depressive symptoms in children of parents with HIV/AIDS. The overall intervention effect size (Cohen’s *d*) was 1.298 and 1.100 for depressive and anxiety symptoms, respectively. Publication bias and exploratory moderating effects of study design (RCT vs. pre–post intervention trials), study location, and intervention levels were also analyzed.

**Conclusion:**

Future studies reporting the detailed outcome data, which could be used for research integration, are warranted. Further research should also focus on the implementation of evidence-based interventions sensitive to the target population in a developmentally appropriate manner.

## Background

The negative influences of HIV are not limited to adults, but they also extend to their younger family members [1–3]. These include HIV-infected or -exposed children as well as seronegative children affected by parental HIV/AIDS [4, 5]. Here, we focused on seronegative children affected by parental HIV/AIDS (i.e., those who were HIV-free and had lost at least one of their parents to HIV/AIDS or were still living with their parents who were infected with HIV). These children can demonstrate more negative developmental outcomes and have higher mental health risk in than do children from HIV-free families [3, 6]. Children affected by parental HIV/AIDS are more likely to have problematic behaviors, such as significant withdrawal, lack of concentration, and delinquency [7, 8]. They are also more likely to have negative feelings, such as high depression and anxiety levels and low self-esteem levels [9, 10]. Compared with children affected by parental cancer or any other potentially lethal and progressive diagnoses, children affected by parental HIV/AIDS experience HIV-related stigma and social isolation, which significantly and negatively affects their mental health and well-being [2, 11].

Studies have been conducted to determine methods to reduce such poor outcomes among children affected by parental HIV/AIDS [1, 12]. Many interventions aimed to help children affected by parental HIV/AIDS have been applied in the past decades, including psychological intervention, social support programs, and physical therapy [13]. Multiple strategies have been employed in these interventions, such as family strengthening strategies, community support services, and self-improvement programs. To identify an efficacious intervention for these children, assessing the magnitudes of the intervention effects is crucial. In a systematic review [13], 17 psychosocial intervention studies were examined to determine the efficacy of these interventions for improving the psychosocial well-being of children affected by parental HIV/AIDS. This review indicated that 15 of 17 intervention studies reported significant effects [12–26], but two reported null intervention effects [27, 28]. Furthermore, this review supported the efficacy of the interventions in improving not only the general psychological well-being but also the social adjustment and school performance of the participants. However, the intervention effects were not quantified, partially because of their broad focus on various intervention outcomes. Although the breadth of the review provided the most comprehensive understanding of the effects of psychosocial interventions in children affected by parental HIV/AIDS, it could not provide detailed, focused, and quantified findings regarding the intervention effects in specific outcomes.

The current study focused on depressive and anxiety symptoms (two important mental health outcomes) and applied meta-analysis to quantify the effects of psychosocial interventions. These interventions emphasized on social or psychological factors, rather than biological factors, as the targets. These interventions can be delivered at the individual, family, group, or community level. We hypothesized that the psychosocial interventions demonstrate significant effects toward the reduction of depressive and anxiety symptoms in children affected by parental HIV/AIDS.

## Method

### Search and selection

This review retrieved interventions studies targeting depression, anxiety, or both, published from 1985 to 2017. Our literature search was conducted at two timepoints. The first search was in February 2015, where we conducted a comprehensive search of electronic databases, including MEDLINE, PsycINFO, and Web of Science using a Boolean searching strategy, to identify studies based on interventions in children affected by parental HIV/AIDS reported in English. We cross referenced the standardized search terms, reflecting three constructs: (a) HIV/AIDS, (b) intervention/prevention, and (c) children/adolescents/teenagers/youths. First, we located all the intervention studies regardless the specific intervention outcomes to have a general understanding of interventions toward children affected by HIV/AIDS. In the search process, we first searched PsycINFO and MEDLINE in EBSCO HOST database by using the Boolean phrase “TI (HIV OR AIDS) AND TI (Intervention OR Prevent*) AND TI (child* or adolescent* or teenage* or youth*)” and found 1296 and 680 articles, respectively; after removing the duplicates, we had a total of 1584 articles from the two databases. We then searched Web of Science by using the Boolean phrase “TI = (HIV OR AIDS) AND TI = (Intervention OR Prevent*) AND TI = (Child* OR Adolescent* OR Teenage* OR Youth*)” and found 1034 articles. Next, after all the articles from the three databases were combined and duplicates were excluded, 1588 articles, which remained, were screened manually. After excluding articles that did not focus on children affected by HIV/AIDS, 39 articles remained for further screening. The second literature search was performed in December 2017 with the same search strategy to identify intervention studies that reported outcomes on depression or anxiety published from February 2015 to December 2017, but no additional studies were identified. We also performed a manual search of the cited references in the review articles regarding children affected by parent HIV and found no additional articles.

Intervention studies were included if they (a) included psychosocial interventions that reported the outcomes on depression or anxiety and (b) were randomized controlled trials (RCTs) or pre–post intervention trials. We did not include the studies on children who are infected with HIV because health issues in this population were beyond the scope of this article. If multiple articles were published based on the same intervention study, we selected the one with the largest sample size as the primary citation. Two research assistants independently assessed the eligibility of the included articles, and there were no disagreements between them.

**Fig. 1:**
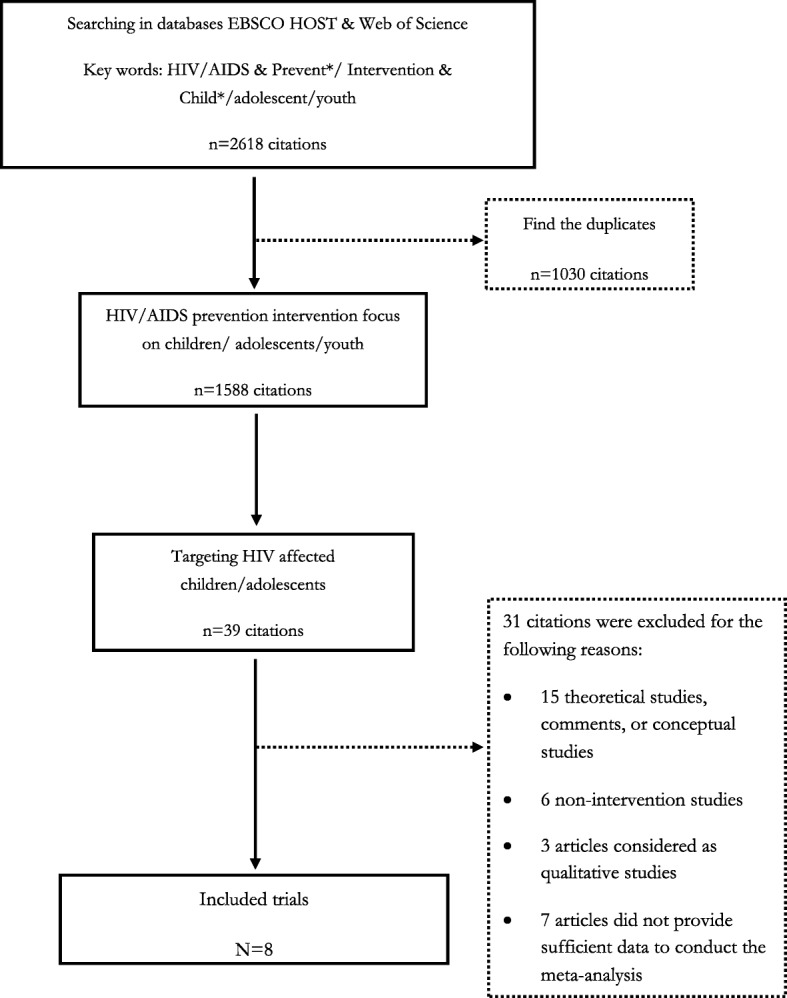
Flow Chart of literature search and study coding

Finally, 15 studies met our eligibility criteria, but seven of them did not provide sufficient raw data for meta-analysis. We emailed the authors of these seven studies requesting for the raw data but received no reply or were told that they were not able to locate the raw data. Consequently, this review included eight studies reporting detailed outcome data required for meta-analysis. Our literature search process is illustrated in Fig. 1.

### Quality assessment

Information regarding eligible interventions was independently abstracted by two trained research assistants and the first author of the current article. The risk of bias was assessed using the components recommended by the Cochrane Collaboration [29], which contains six aspects: allocation sequence generation; allocation concealment; blinding of participants, staff, and outcome assessors; incomplete outcome data; selective outcome reporting; and other bias sources. Disagreements between the research assistants were resolved through discussion with the first author of this article. In general, the included eight studies were deemed to have a low risk of bias. The detailed evaluation for each study is provided in Table 1.

**Table 1:** Quality assessment of included studies

Study	Random sequence generation	Allocation concealment	Blinding of participants and personnel	Blinding of outcome assessment	Incomplete outcome data addressed	Selective reporting	Other Bias
Murphy et al., 2015 [30]	Low Risk	Low Risk	Low Risk	Low Risk	Middle Risk	Low Risk	Low Risk
Lin et al., 2014 [31]	Low Risk	High Risk	Low Risk	Low Risk	Low Risk	Low Risk	Low Risk
Eloff et al., 2014 [12]	Low Risk	Low Risk	Low Risk	Low Risk	Middle Risk	Low Risk	Low Risk
Rochat et al., 2014 [32]	Low Risk	High Risk	Low Risk	Low Risk	Low Risk	Low Risk	Low Risk
Keypour et al. 2011 [33]	High Risk	High Risk	Low Risk	Low Risk	Low Risk	Low Risk	Middle Risk
Kumakech et al., 2009 [16]	Low Risk	Low Risk	Low Risk	Low Risk	Low Risk	Low Risk	Middle Risk
Kaufman et al., 2013 [34]	High Risk	High Risk	Low Risk	Low Risk	Low Risk	Low Risk	Middle Risk
O’Donnell et al., 2014 [35]	High Risk	High Risk	Low Risk	Low Risk	Low Risk	Low Risk	Low Risk

By using standard forms, each study was abstracted for the intervention information (e.g., location, intervention date, recruitment strategy, and sample size), participants’ characteristics (e.g., age and gender), and intervention features (e.g., intervention level, duration, component, and design). The detailed descriptive summary is presented in Table 2.

**Table 2:** Descriptive summary of the included studies

Study	Location (dates of the study)	Sample Size and Characteristics	Evaluation Design	Intervention Description	Major Findings
Murphy et al., 2015 [30]	Los Angeles, USA	37 mother-child dyads through HIV/AIDS service organization; Child *M* age 10.5 (7–14); Women *M* age 37.6	RCT: Yes, waiting-list design; Comparison: Standard Care; Follow-ups: Baseline, after intervention, Retention: 100%.	Name: Children United with Buddies (CUB); Level: Group; Components: 3 sessions for children, (a) learn concrete coping strategies, especially communication with their mothers living with HIV (MLH); (b) obtain accurate and age-appropriate information on how HIV is not transmitted, and on HIV illness and treatment; and (c) feel less isolated, by providing connection to peers who share similar issues and promoting support for normalization and validation of fears and concerns. MLH attended the first two sessions with children and the third session paralleled; Duration: Weekly session, each session lasts 60–75 min; Deliverer: two pairs of facilitators held master’s degree in psychology	Results showed significant decreases in anxiety and worry for children in the intervention group and increases in happiness and knowledge regarding HIV/AIDS transmission.
Lin et al., 2014 [31]	Central China	*N* = 124, children orphaned by AIDS (COA)	RCT: Yes;Comparison: Standard care; Follow-ups: Baseline, three-month posttest.	This psychological intervention program was designed to specifically help COA process their grief, cope with stress and discrimination, manage emotion, and reduce their psychological distress. Six weekly sessions (1.5–2.0 h) of group intervention focused on processing grief, reducing traumatic symptoms and psychological distress, and fostering hope about the future. Deliverer: a trained postgraduate student with a degree in family therapy	At the three-month post-test, children in both groups (intervention group and control group) reported significant reduction in trauma symptoms and demonstrated similar levels of hope. However, the intervention group reported significantly increased levels of grief processing and decreased levels of trauma symptoms, depression, and threat appraisal.
Eloff et al., 2014 [12]	Tshwane, South Africa	390 HIV+ women attending clinics and their children; Child *M* age 8.4 (6–10); Women *M* age 33.1;	RCT: Yes; Comparison: Standard Care; Follow-ups: IP, 6-months,12-months, 18-months; Retention: 84.6%.	Name: Parent-Child Group Intervention; Level: Group; Components: 14 separated interventions for mothers (on living with HIV and parenting) and children (on building self-esteem, enhancing interpersonal and practical life skills); 10 joint sessions (on healthy parent-child interaction); Duration: weekly session, each lasting 75 min; Deliverer: Community care workers.	Intervention children reported a temporary increase in anxiety but did not report differences in depression. However, boys tend to gain greater benefit from the intervention than girls in reducing depression.
Rochat et al., 2014 [32]	South Africa	291 mothers living with HIV and their HIV-uninfected children aged 6–10 years	RCT: No, pre-post design; Comparison: Standard care; Follow-ups: Baseline, after intervention.	Name: Family centered, maternal HIV disclosure interventions; Level: Individual; Components: 1) a pre-disclosure stage when the counselor worked with the mother to prepare and train her towards disclosure; 2) a post-disclosure stage, when the mother was counseled on health promotion and custody planning. Duration: 6 sessions, over a six to eight-week period. Mothers attended all the sessions and children joined in the second and last sessions. 1–2 h in session 1,2, and 5, 2–3 h in sessions 3 and 4, less than 1 h in session 6. Deliverer: Lay counsellor	There was a significant decrease in CBCL total scores, including significant decreases in anxious-depressed, withdrawn-depressed. A significant moderating effect of disclosure type was revealed on withdrawn-depressed syndrome scores; thus, when compared to those who fully disclosed, children of mothers who partially disclosed had a greater decrease in withdrawn-depressed syndrome scores after intervention.
Keypour et al. 2011 [33]	Iran	15 adolescents (age = 13–18) and 15 HIV+ parents	RCT: No, pre-post design; Comparison: Standard care; Follow-ups: Baseline; after intervention, 3 months.	Name: Cognitive behavioral stress management training; Level: Group; Components: Adolescents interventions focuses on awareness about stress, relaxation, unhelpful thoughts, cognitive reconstruction, problem solving, assertiveness skill training, anger and time management (8 weekly sessions, 90 min each; 3 educational sessions for parents (parallel to first, fourth and eighth children’s session); Deliver: A child and adolescent psychiatrist	The training improved adolescents’ emotional problems, including anxiety and depression.
Kumakech et al., 2009 [16]	Mbarara District of southwestern Uganda	326 Children (age = 10–15) who have lost one or both parents due to AIDS were selected using a multi-stage sampling procedure	RCT: Yes; Comparison: Standard care; Follow-ups: Baseline, 10 weeks after intervention; Retention: 91.4%.	Name: Peer-group support intervention; Level: Group; Components: a) introduction and relationships building; b) trust building and group discussion of problem solving, fears of orphanhood, and sources of satisfaction; c) self-esteem raising exercises. Duration: 16 sessions (over 10 weeks), each one lasts approximately 1 h; Deliverer: selected primary school teachers (supervised by a researcher and a counselor).	The peer-group support intervention has a significant impact on anxiety, depression, and anger among AIDS orphans.
Kaufman et al., 2013 [34]	China	39 children orphaned by AIDS	RCT: NO, Pre-post design; Comparison: Standard care; Follow ups: Baseline; 6- and 12- month follow-up;	Name: Community-based mental health counseling. Level: Individual, Components: To improve communication skills for children and caretakers and reinforce children’s self-esteem including two joint sessions with caretakers. Two rounds of counseling (six sessions in round one, four sessions in round two, once a week for 90 min) were carried out over a one-year. Deliverer: Trained community workers	There was a statistically significant improvement for the children on anxiety, but there was no statistically significant improvement on depression, with greatest gains immediately following the intervention.
O’Donnell et al., 2014 [35]	Tanzania, East Africa	64 orphaned children	RCT: NO; Pre-post; Follow-ups: Baseline, 3-month, and 12-month follow-up.	Name: group-based trauma-focused cognitive behavior therapy (TF-CBT) Level: Group, Components: a) a foundation for understanding how loss affects children and taught relaxation and coping skills (session 1–3). b) The trauma narrative (TN) (e.g. talking about memory and sharing in session 5–8) and processing (discuss feeling and support children). The last sessions (9–12) addressed grief-specific elements, each including a conjoint child–guardian activity. Deliverer: Lay counselors with no prior mental health experiences.	Results: Children had reduced depression symptoms by the end of treatment, with improvements sustained at 3 and 12 months after treatment.

### Data analysis

The analyses were conducted using Comprehensive Meta-Analysis (version 2.0). We conducted the analyses on the efficacy of the interventions toward depression and anxiety separately. Effect size (Cohen’s *d*) was calculated [36] by using random-effects model of DerSimonian and Laird [37] for meta-analysis. A Cohen’s *d* of .2, .5, and .8 represents a small, medium, and large effect size, respectively [36, 38]. For pre–post intervention trials, we calculated the mean differences of the scores between the baseline (before the interventions) and the first follow-up assessment after the interventions. Similarly, for the RCT studies, we obtained data from the first follow-up of each study and analyzed the difference in their scores before and after the intervention for both intervention and control groups, with the pooled standard deviations [39]. The pooled standard deviations for each group of the trials were obtained through point estimate for each single trial weighted by the inverse of the variance (1/SE^2^) [36]. To assess study heterogeneity, *I*^*2*^ was examined. The *I*^*2*^ qualifies the proportion of total variance across studies caused by real difference between trials, rather than that by chance. *I*^*2*^ lt; 25% indicates low observed heterogeneity, whereas *I*^*2*^ > 75% indicates high observed heterogeneity [40].

To analyze the publication bias and possibility of small-study bias, funnel plots were tested visually. Egger weighted regression test [41] and Begg and Mazumdar [42] correlation test were also performed. We also examined three potential moderators of the intervention efficacy: intervention design, study location, and intervention levels. The intervention design of the included studies was either pre–post intervention trial or RCT. Study locations included the United States, China, Africa, and Iran. There were two intervention levels of the included studies, namely child only level and child–caregiver dyadic level. Moderation analysis was then performed to examine the potential difference between the subgroups according to a *Q* test based on analysis of variance. The intervention effect between the tested subgroups was considered to be different when the *p* value was <.05 [43].

## Results

### Intervention characteristics

Four interventions were conducted in Africa [12, 16, 32, 35], one in the United States [30], two in China [31, 34], and one in Iran [33]. Moreover, four interventions were RCTs [12, 16, 30, 31] and four were pre–post intervention trials. Four studies measured the outcomes in multiple follow-ups, with the longest follow-up duration being 18 months [12]. Other studies included a single assessment immediately [30, 32], 10 weeks [16], or 3 months [31] after the completion of intervention sessions.

The interventions were delivered by mental health professionals (i.e., facilitators with major in psychology and psychiatrists) [30, 31, 33] or by mental health paraprofessionals (i.e., trained community care workers, lay counselors, and school teachers). All reviewed intervention trials had multiple sessions with the average time of a session being approximately 1–1.5 h. The shortest durations of the intervention was about three sessions [30], and the longest duration of the intervention was about 24 sessions [12], respecitively. Most intervention sessions were delivered weekly. One study delivered 6 sessions over 6–8 weeks [32] and another study delivered 16 over 10 weeks [16].

### Intervention levels and components

Family system was considered a crucial level of intervention and design by four studies; therefore, parallel and joint sessions of the interventions for children and their caregivers, including HIV-positive or -negative parents, were designed and delivered [12, 30, 32, 33]. Most of the joint sessions focused on positive communication and mutual support between children and their HIV-positive parents. The other four studies designed the interventions only for children. The types of the interventions included group-based trauma-focused cognitive-behavioral therapy [35]; group-based social support promoting therapy [16]; individual mental health counseling [34]; group-based grief-processing [31]; cognitive-behavioral stress management training [33]; children united with buddies program [30]; family-centered, maternal HIV disclosure interventions [32]; and positive parenting intervention [12].

### Effect size of intervention regarding anxiety

Eight studies were reviewed, of which six reported anxiety. Cohen’s *d* of the interventions regarding anxiety was 1.100 (95% CI = [0.351, 1.849], *p* = .004), indicating a significantly large effect size (Table 3). According to the results of heterogeneity test (*I*^*2*^ = 95.613, *τ*^2^ = .799), the between-studies variability in effect sizes were due to a large amount of heterogeneity, rather than a random error. We then explored the potential heterogeneity factors, namely intervention design, study location, and intervention levels, through moderation analysis.

**Table 3:**
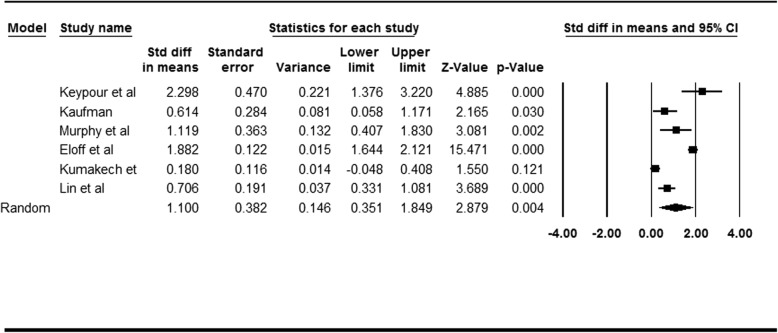
Overall effect size for 6 included studies of psychological interventions towards anxiety among children affected by HIV/AIDS (*N* = 6)

#### Moderating effect of intervention design

Of the selected studies, four were RCTs and two were pre–post intervention trials without a control group. The intervention design was a significant moderator (*Q* (1) = 0.212, *p* = .01). The intervention effects toward anxiety were nonconclusive in the pre–post intervention trails (*d* = 1.414, 95% CI = [− 0.233, 3.062], *p* = .093), but significant in the RCTs (*d* = 0.969, 95% CI = [0.024, 1.913], *p* = .044).

#### Moderating effect of study location

Two studies were conducted in Africa, two in China, and once each in Iran and the United States. The location was a significant moderator (*Q* (3) = 11.176, *p* < .001). The intervention effects were significant for the studies in China (*d* = 0.677, 95% CI = [0.366, 0.988], *p* < .001), Iran (*d* = 2.298, 95% CI = [1.376, 3.220], *p* < .001), and the United States (*d* = 1.119, 95% CI = [0.407, 1.830], *p* = .002). The intervention effects were nonconclusive for the studies in Africa (*d* = 1.031, 95% CI = [− 0.637, 2.699], *p* = .226).

#### Moderating effect of intervention levels

Three studies recruited both children and their caregivers as participants, whereas the other three recruited only children affected by HIV/AIDS. The intervention level was a significant moderator (*Q* (1) = 14.367, *p* < .001). The efficacy of intervention was more significant in studies targeting both children and family members (*d* = 1.754, 95% CI = [1.207, 2.300], *p* < .001) than those studies targeting only children (*d* = 0.462, 95% CI = [0.079, 0.846], *p* = .018).

#### Publication bias assessment

**Fig. 2:**
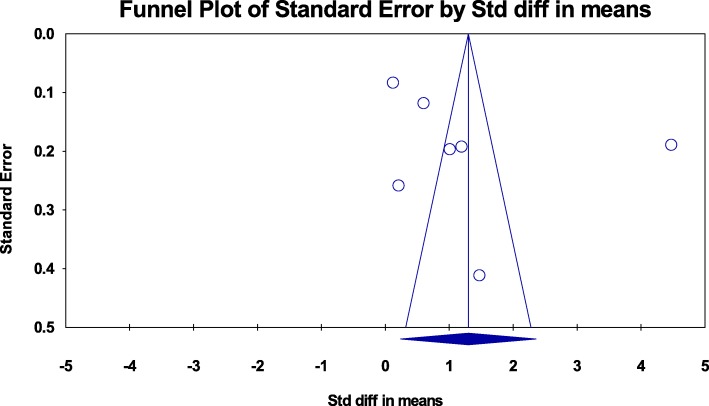
Funnel plot for publication bias assessment (intervention Efficacy of studies reporting results towards depression). *Note.* Egger’s test: *p* = .808; Begg and Mazumda’s test: *p* = .764

The funnel plots were nonsignificant for Egger test (*p* = .284) and Begg and Mazumdar test (*p* = .260), indicating the absence of anxiety-related publication bias in the reviewed studies (Fig. 2).

### Effect size for studies reporting intervention efficacy toward depression

Eight studies were reviewed; of them, seven reported depression outcomes. Cohen’s *d* of the interventions toward depression was 1.298 (95% CI = [0.240, 2.357], *p* = .016), indicating significant and large effect size (Table 4). According to the heterogeneity test (*I*^*2*^ = 98.687, *τ*^2^ = 1.990), the between-study variability in the effect sizes were due to a large amount of heterogeneity. We again explored the potential heterogeneity factors, namely intervention design, study location, and intervention level, using moderation analysis.

**Table 4:**
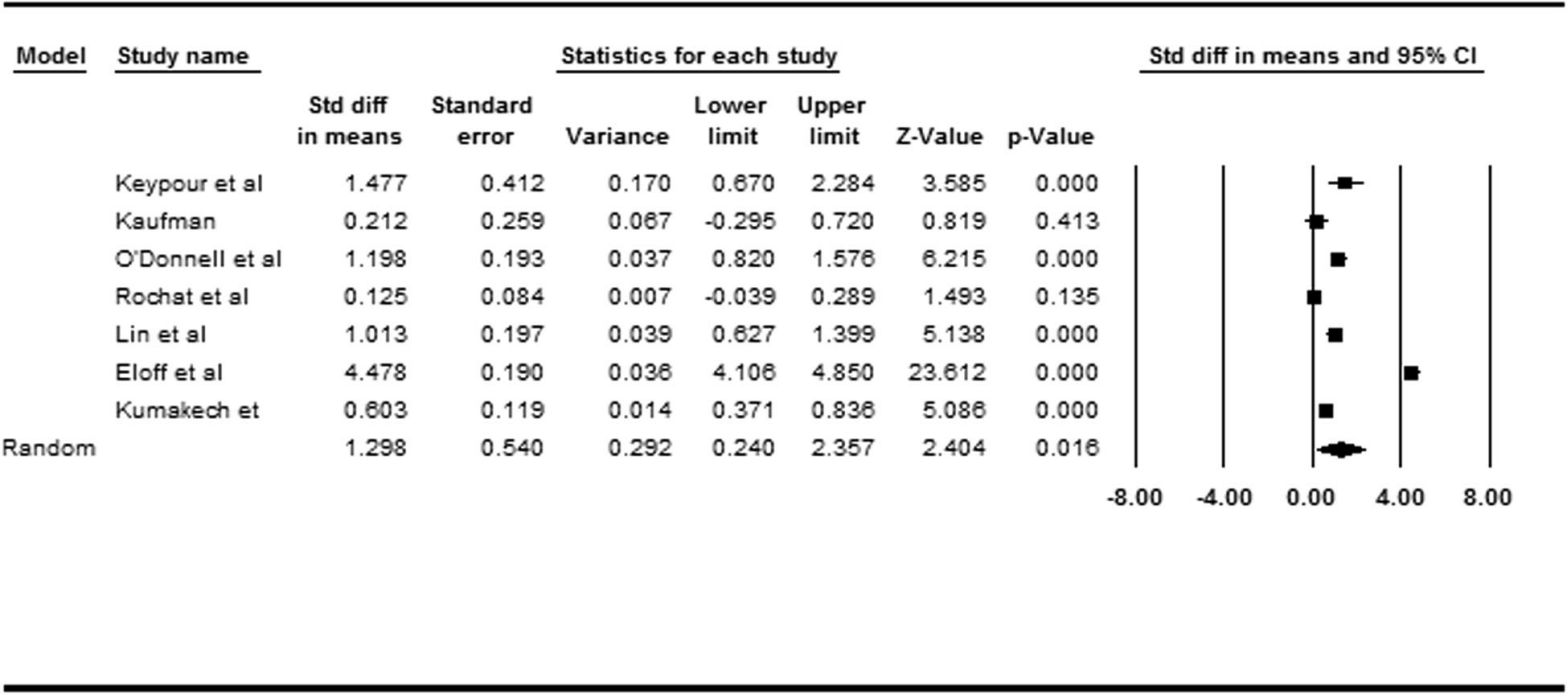
Overall effect size for 7 designed studies of psychological interventions towards depression among children affected by HIV/AIDS (*N* = 7)

#### Moderating effect of intervention design

Four reviewed studies were pre–post intervention trials without a control group and three were RCTs. The intervention design was a significant moderator (*Q* (1) = 1.140, *p* = .014). The intervention effects toward depression were significant for the pre–post intervention trials (*d* = 0.705, 95% CI = [0.039, 1.371], *p* = .038), but nonconclusive for the RCTs (*d* = 2.029, 95% CI = [− 0.309, 4.367], *p* = .089).

#### Moderating effect of study location

Four of the studies were conducted in Africa, two in China, and one in Iran. The location was a significant moderator (*Q* (2) = 2.584, *p* < .001). The intervention effects toward depression were significant for studies conducted in Iran (*d* = 1.477, 95% CI = [0.670, 2.284], *p* < .001), but nonconclusive for studies conducted in China (*d* = 0.630, 95% CI = [− 0.154, 1.414], *p* = 0.115) and Africa (*d* = 1.595, 95% CI = [− 0.006, 3.196], *p* = .051).

#### Moderating effect of intervention level

Three studies targeted both caregivers and children, and four targeted only children affected by parental HIV/AIDS. The intervention level was a significant moderator (*Q* (1) = 0.591, *p* = .442). The intervention effects toward depression were significant for studies targeting only children (*d* = 0.772, 95% CI = [0.395, 1.148], *p* < .001), but were nonconclusive for studies targeting both caregivers and children (*d* = 2.027, 95% CI = [− 1.153, 5.208], *p* = .211).

#### Publication bias assessment

**Fig. 3:**
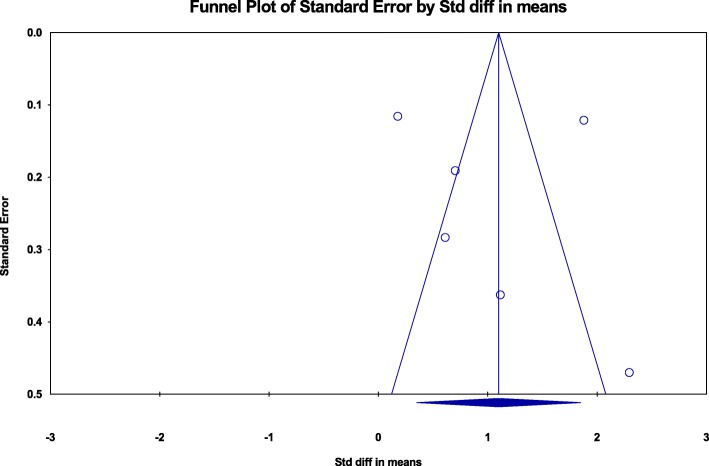
Funnel plot for publication bias assessment (intervention Efficacy of studies reporting results towards anxiety). *Note.* Egger’s test: *p* = .284; Begg and Mazumda’s test: *p* = .260

The examination of funnel plot presented in Fig. 3 revealed the non-significance in the Egger test (*p* = .808) and the Begg and Mazumdar rank correlation test (*p* = .764), suggesting a low risk of publication bias for the reviewed studies regarding depression.

## Discussion

A set of meta-analyses were performed to examine the efficacy of various psychosocial interventions for the reduction of depression and anxiety in children affected by parental HIV/AIDS. Although the interventions had great diversity with respect to design and components, they demonstrated consistent positive effect sizes for the efficacy of various psychosocial interventions for depression and anxiety. For depression and anxiety the overall effect sizes were 1.298 and 1.100, respectively. Our study findings are consistent with those of a previous systematic review [13]; they are a quantitative extension of conclusion of previous studies in terms of the intervention effects for reducing depressive and anxiety symptoms in children affected by parental HIV/AIDS.

The analyses of moderating effects of intervention design, study location, and intervention levels provided some preliminary and exploratory information regarding the intervention effectiveness in different contexts. Moderation analysis on study regions suggested that psychosocial interventions appeared to be less effective in resource-limited countries (i.e., those in Africa). The participants in the African countries might still be in need of food, shelters, clean water, and other basic living facilities. Moreover, in these countries, HIV/AIDS usually co-occurs with poverty and other diseases (e.g., tuberculosis and malaria) [44]. Our analyses suggested that multilevel interventions, including psychosocial interventions along with poverty-prevention and disease-control programs, may support children affected by parental HIV/AIDS more efficiently. When resources are extremely limited, a single-level psychosocial intervention do not yield sufficient effect on mental health [12, 45]. In the long term, the basic living needs should be satisfied first in order to develop psychological resilience to cope with other problems at cognitive and emotional levels.

We found some inconsistencies in the results in terms of the moderating effects of intervention levels through the meta-analysis. The dyadic interventions were more effective on anxiety but less effective on depressive symptoms compared with individual-level interventions. Although only three dyadic intervention studies were included in this meta-analysis, one of the dyadic intervention studies recruited a large sample (*N* = 390 mother–child pairs) and revealed less conclusive intervention effect on depression [12]. The results of this study might have caused bias in the meta-anlaysis results. Caution should be excercised when interpreting the dyadic interventions for reducing depressive symptoms in children, with nonsignificant effects, given that interventions at multiple levels in socioecological system of children have been suggested [2]. Furthermore, our findings warrant replication for the large but nonsignificant Cohen’s *d* (2.027 vs. 0.772) in interventions with dyadic level. Future meta-analyses including more studies may provide more informative evidence regarding the efficacy of dyadic interventions in reducing depressive symptoms.

The effects of interventions in the RCTs for reducing anxiety were found to be significant, which was as expected. However, the effects of interventions in the RCTs for improving depressive symptoms were less conclusive. The non-significance in results for depression in the study of Eloff et al. [12] might have weighed more in our meta-analysis results due to the larger sample size. Eloff et al. [12] suggested that mothers reported significant decrease in depression, and among the children, boys reported a significant decrease in depression but girls did not. Gender might moderate the intervention efficacy on depression. However, all the other included studies did not report such gender-specific data; therefore, we could not conduct the meta-analysis of the gender difference in intervention effects based on the current selected studies. Future empirical studies may focus on identifying specific protective factors that could help children affected by HIV/AIDS with different genders to improve their psychological well-being. Eloff et al. [12] also suggested that children presented improved adaptive behaviors and decreased externalizing behavior problems. Longer follow-ups might be needed to explore the emotional changes and internalizing behaviors of children in more detail. We only included the first follow-ups in our meta-analysis and could not examine the long-term effects of psychosocial interventions on depressive symptoms.

The current study has some limitations. First, we included only peer-reviewed articles published in English and excluded those in other languages and those on government reports and NGO program evaluation reports; these excluded studies warrant consideration in further investigation. Second, we focused only on two internalizing problems, namely depression and anxiety, which were included in most of the reviewed studies. However, we noted that different intervention studies had reported many other outcomes, such as substance abuse, delinquency, school performance, and adaptive functioning. Further research may focus on other developmental outcomes, when there is sufficient number of intervention studies that focused on these outcomes. Third, we focused only on children affected by parental HIV/AIDS. Although our approach is well-justified based on the scope of the current research, studies reporting interventions targeting children with HIV infection have also been reported. Thus, meta-analysis on various outcomes (e.g., cognitive ability) in children with HIV infection may provide valuable information to policy-makers in terms of positive child development. Finally, moderation analyses on intervention design and levels were less explainable because of the limited number of included studies and the inconsistent patterns in the findings. Additional studies validating the current findings or having a meaningful interpretation are warranted.

## Conclusion

In conclusion, psychosocial interventions were generally effective in reducing depression and anxiety in children affected by parental HIV/AIDS. These results were consistent with the recently published systematic review, which supported the efficacy of the psychosocial intervention in general for improving psychological well-being [13]. Since the study of King, De Silva, Stein, and Patel [46], researchers and practitioners have made tremendous efforts in providing psychosocial support to children affected by HIV/AIDS. However, some knowledge gap remains in this area of research and practice. Future intervention studies may improve on the rigorousness of the intervention design, such as follow-up timing and frequency, intervention level, and target population (e.g., boys vs. girls). In addition, to facilitate research integration in the future, valid reports on study procedure and detailed outcome data are required. A standard report of interventions with detailed data for future research synthesis and meta-analysis is warranted. Many of the intervention studies did not adequately report the outcome data; therefore, the total number of studies (*n* = 8) included in the final analyses was very small and this may have reduced the validity of the calculated effect sizes. Moreover, the inconsistent results of the moderating effects by intervention levels may be due to the limited number of studies reviewed. Additional studies with adequate raw data (e.g., raw means and standard deviations) presenting checklists and statements from consolidated standards of reporting randomized trials [47] would be highly beneficial for further research in this area.

## Data Availability

The datasets used and/or analyzed during the current study available from the corresponding author on reasonable request. The data can be obtained by emailing: peilianchi@um.edu.mo; peilianchi@gmail.com.

## Abbreviations

AIDS: Acquired Immune Deficiency Syndrome
HIV: Human Immunodeficiency Virus
NGO: Non-Government Organization
RCT: Randomized Controlled Trials
